# The Treatment of Hysteria

**Published:** 1919

**Authors:** 


***The Treatment of Hysteria.*** By JULIAN MAST WOLFSOHN, Captain, M. C., U. S. A. *Journal of American Medical Association.*

Wolfsohn gives an analytical review of 570 cases of hysteria which he has treated by a rapid re-educative method.

The various types encountered include mutism, deafness, aphonia, stammering, blepharospasm, blindness, monoplegia, paraplegia, tics, astasia abasia, staso basophobia, dancing gaits, hemiplegias, tremors, photophobias and platysma spasm. Of these there were only twelve cases where an external wound complicated the picture. A rapid cure was accomplished in about 90 o/o of the cases.

He divides the treatment of hysteria as practiced at present into the slow and the rapid methods. The former include (1) purely re-educative methods, such as massage, lessons in speaking for stammerers, etc.; (2) psychotherapy, especially psycho-analysis, often accomplishes much, but it is impracticable on a large scale because of the lack of experts. It is also too slow from a military standpoint. The rapid methods include: (1) hypnotism; (2) general anesthesia; (3) continuous bath; and (4) strong suggestion supplemented by a mechanical agent, such as electricity.

He details the treatment in each type of hysterical symptom and concludes his article with the following rules: -

1. Study each case fully. Take a complete family and personal history, especially for previous nervousness or similar attacks, even though the character of the affection appears evident.

2. Study each case psychologically, before instituting treatment. This can take the form of a psycho-analysis in the cases in which emotivity plays a prominent part. Encourage the patient to talk of himself and thus gauge his mental condition.

3. Produce an atmosphere of cure in the wards or office. This is best accomplished by contact with cured cases. Confident optimistic atmosphere has helped to cure more hysterical symptoms than all the mechanical apparatuses.

4. The patient must seek the cure.

5. When the patient resists the cure, this must be broken down by strong persuasion, long continued, or by faradism.

6. The physician must, at all times, be master of the situation.

He must have a ready response to all questions put to him by the patient.

7. Do not discontinue the treatment until the cure is accomplished.

8. Let the patient into your confidence, explain to him in simple terms how his symptom is caused and how you propose to cure it immediately.

9. Be absolutely sure of your diagnosis before beginning treatment.

10. A quiet firm sympathy, together with an air of supreme confidence, is essential for the " air of recovery " in the treatment room.

11. Hysteria is often promoted in the ward by mental contagion. If a severe case is admitted it is best to isolate it for a short while and thus prevent this contagion.

12. The earlier the treatment is instituted after the occurrence of the symptom, the easier and more complete the cure. The fixation of the idea is thus prevented.

13. During the treatment of a paralyzed member, watch for the slightest voluntary movement of the limb on the part of the patient, and use that for the point for further suggestion.

14. The method of treatment varies with the mentality of the patient, the desire for recovery, the degree of education and his social level.

(*a*) To the cultured and intelligent, frankness should be the keynote. Suggestion often suffices for the cure.

(*b*) To the uneducated, simple suggestion plus a material method is usually necessary.

15. To avoid the distraction of the patient during treatment, all extraneous noises and movement should be eliminated.

16. The physician must possess a strong will power. He cannot convince unless he himself is convinced. He must display kindness, but his commands must be full of authority.

17. Results of treatment depend on the physician who plays the leading part. The patients are cured when they find their master.

18. Do not lose your temper. Have patience.

19. Select the best time for treatment and if not successful after a time, cease, and recommence at a more favorable opportunity, explaining to the patient that rest is the cause for stopping. Meanwhile he should be completely isolated.

20. Do not give fresh suggestions to the patient nor threaten him unless he is known to be a malingerer.

21. The ease of durability is largely dependent upon the duration of the symptoms, and the nature and number of previous treatments.

22. Contractures are more persistent than paralyses.

23. Strict military discipline and regular outdoor work are the necessary after-cure of these patients.

24. Make the disease an unprofitable one for the patient.

25. The cure of a hysterical symptom is a mental combat between the physician and the patient, in which the victory is on the side of the physician. This is the secret of psychotherapy.

26. The patient should be observed for a few weeks after the cure.

27. The most potent causes of failure to cure are illwill of the patient, unfavorable surroundings, and *mistakes in diagnosis*.

With these considerations as working hypotheses, any physician should be able to treat hysterical disorders successfully, but without them failure stares him in the face.

